# *Porphyromonas gingivalis* OMVs inhibit osteogenic differentiation of BMSCs via SAA3/TLR4/MyD88/NF-κB axis

**DOI:** 10.1080/20002297.2025.2540823

**Published:** 2025-08-08

**Authors:** Yongyong Yan, Haiyan Wang, Huizhi Deng, Haokun He, Qing Ge, Jun Zha, Jun Chen, Qing Zhang, Haiyan Deng, Gang Wu, Richard T. Jaspers, Janak L. Pathak

**Affiliations:** a School and Hospital of Stomatology, Guangdong Engineering Research Center of Oral Restoration and Reconstruction & Guangzhou Key Laboratory of Basic and Applied Research of Oral Regenerative Medicine, Guangzhou Medical University, Guangzhou, China; b Laboratory for Myology, Department of Human Movement Sciences, Faculty of Behavioural and Movement Sciences, Vrije Universiteit Amsterdam, Amsterdam Movement Sciences, Amsterdam, the Netherlands; c The First Affiliated Hospital of Guangdong Pharmaceutical University, Guangzhou, China; d Savid School of Stomatology, Hangzhou Medical College, Hangzhou, China; e Department of Oral and Maxillofacial Surgery, Leiden University Medical Center (LUMC), Leiden, the Netherlands

**Keywords:** *P. gingivalis*, outer membrane vesicles, serum amyloid a, osteoblast differentiation, BMSCs, Periodontitis

## Abstract

**Backgrounds:**

Periodontitis-induced alveolar bone loss is a primary cause of tooth loss. *Porphyromonas gingivalis* (*P. gingivalis*) is the primary pathogenic bacterium of periodontitis. Outer membrane vesicles (OMVs) derived from *P. gingivalis* (*P.g*-OMVs) contain various bioactive molecules, and several studies have suggested that *P.g*-OMVs may participate in alveolar bone loss caused by periodontitis.

**Materials and Methods:**

*P.g*-OMVs were isolated and characterized. The effect of *P.g*-OMVs on BMSCs proliferation and osteogenic differentiation was analyzed. High-throughput sequencing, RT-qPCR, and Western blot analysis were performed in BMSCs to unravel the underlying molecular mechanism.

**Results:**

*P.g*-OMVs promoted proliferation but inhibited osteogenic differentiation of BMSCs. High-throughput sequencing results showed that serum amyloid A (SAA), especially SAA3, was robustly upregulated in *P.g*-OMVs-treated BMSCs. Upregulated SAA3 promoted TLR4, MyD88, and NF-κB p65 and inhibited osteogenic differentiation of *P.g*-OMVs-treated BMSCs. The knockdown of SAA3 in BMSCs downregulated *P.g*-OMVs-induced TLR4, MyD88, and NF-κB p65 and rescued *P.g*-OMVs-inhibited osteogenic differentiation.

**Conclusions:**

Our results indicate that *P.g*-OMVs inhibit osteogenic differentiation of BMSCs via the SAA3-mediated TLR4/MyD88/NF-κB axis, providing novel targets for the treatment of periodontitis-induced alveolar bone loss.

## Introduction

Periodontitis is caused by pathogenic oral microbiota-triggered chronic infectious inflammation of periodontal support tissues. Periodontitis causes degeneration of the supporting tissues of teeth, loss of periodontal attachment, and tooth loss. *Porphyromonas gingivalis* (*P. gingivalis*) is a gram-negative, anaerobic, rod-shaped bacterium that is detected in 85.75% of subgingival biofilm of patients with chronic periodontitis and has been confirmed as one of the major pathogens of periodontitis [1]. *P. gingivalis* synthesizes virulence factors such as lipopolysaccharides (LPS) and fimbriae, which not only enhance bacterial invasiveness and pathogenicity but also interact with adjacent gingival cells, including immune cells, fibroblasts, and stem cells, leading to an increased release of pro-inflammatory mediators that ultimately result in damage to gingival tissues and periodontal ligaments [2]. *P. gingivalis* and its metabolites inhibit the osteogenic differentiation of precursor cells and promote osteoclastogenesis, causing alveolar bone loss [3]. However, the exact mode of action of *P. gingivalis* during osteogenic differentiation of precursor cells remains unclear.

Researchers have recently revealed that bacterial outer membrane vesicles are the critical pathogenic mediator during bacterial infection [4]. Bacterial OMVs comprise double-layered spherical membrane structures of about 50–250 nm diameter that emerge from gram-negative bacteria’s cell walls during growth and then separate from their peptidoglycans. OMVs comprise outer membrane proteins, LPS, phospholipids, DNA, and some periplasmic components wrapped by the outer membrane during formation [5]. Bacterial OMVs participate in adapting to stress, acquiring nutrients, and communicating with host cells and other bacteria [6]. Many enriched components related to OMVs are pathogenic factors that cause host cell damage, immune system evasion, host cell invasion, and antibiotic resistance [7]. The production of OMVs derived from *P. gingivalis* (*P.g*-OMVs) was first reported in 1985, but their physiological function and pathogenesis remain unclear [8]. In recent years, increasing evidence suggests that *P.g*-OMVs have more ubstantial virulence than parental bacteria and play an essential role in the pathogenesis of periodontitis [9]. Fan et al. showed that *P.g*-OMVs regulate apoptosis in human periodontal ligament cells (hPDLCs) through sRNA45033 targeting CBX5 [10]. Yuta et al. showed *P.g*-OMVs activated ERK1/2, JNK, p38 MAPK, STING, and NF-κB signaling pathways, resulting in increased interleukin (IL)-6 and IL-8 expression in human gingival epithelial cells [11]. An increasing body of literature indicates that *P.g*-OMVs can deliver *P. gingivalis* virulence factors to host cells in distant organs, such as the brain [12,13] and liver [14]. BMSCs serve as critical seed cells for bone regeneration, including but not limited to alveolar bone regeneration. However, the role of *P.g*-OMVs in the osteogenic differentiation of BMSCs has not been investigated yet.

Serum amyloid A (SAA) is mainly produced in the liver, and its production increases in response to bacterial/viral infections, autoimmune diseases, and metastatic cancers [15]. SAA has been highly conserved throughout evolution and implicated in adaptive and innate immunity [16]. New research points to SAA as a damage-associated molecular pattern (DAMP) protein, regulated in the case of acute injuries such as periodontitis [17,18]. A recent review has outlined several mechanisms by which SAA amplifies inflammation, including its ability to engage surface receptors such as Toll-like receptors (TLRs), the receptor for advanced glycation end-products (RAGE), and formyl peptide receptor 2 (FPR2), thereby activating downstream inflammatory signaling cascades [19].

This study investigated the role of *P.g*-OMVs in osteogenic differentiation of BMSCs and the underlying mechanism. *P.g*-OMVs inhibited osteogenic differentiation of BMSCs via the SAA3/TLR4/myeloid differentiation primary response protein 88 (MyD88)/nuclear factor Kappa B (NF-κB) axis. Pathogenic bacteria-mediated inhibition of osteogenic differentiation of precursor cells is the key contributor to alveolar bone loss during periodontitis [20–22]. Our findings indicated that inhibition of *P.g*-OMVs release and targeting SAA3-mediated TLR4/MyD88/NF-κB axis could be possible strategies to rescue periodontitis-induced alveolar bone loss.

## Materials and methods

### Bacteria and culture conditions

*P. gingivalis* (ATCC 33,277) was grown anaerobically in a Coy anaerobic chamber at 37°C with an atmosphere of 86% nitrogen, 10% carbon dioxide, and 4% hydrogen. The culture medium used was tryptic soy broth supplemented with yeast extract (TSBY), 5% yeast extract, 2% sodium bicarbonate, 7.5 μM hemin, and 3 μM menadione. TSB blood agar plates (BAP) were prepared by adding 5% sheep blood and 1.5% agar to the TSB medium. BAP was then inoculated into 5 mL of TSBY and incubated anaerobically at 37°C for 18–24 h until the early log phase was reached after dilution with TSBY. Every 3 h, 1 mL of bacterial culture was obtained, and bacterial concentration was measured using a spectrophotometer at 600 nm until the bacterial cells reached the stationary phase of growth.

### *P.g*-OMVs preparation

The conditioned medium from *P. gingivalis* log phase culture was transferred to a 50 mL centrifuge tube and centrifuged at 300 × g for 10 min to collect the supernatant. The supernatant was subjected to a series of low-speed centrifugation steps (3,000 × g for 20 min) to discard cellular debris. The supernatant was then filtered through a 0.22 μm filter and diluted with four times the volume of PBS (Gibco, MA, USA). Subsequently, the supernatant was concentrated using centrifugal filters (50 kD, Amicon Ultra, Ireland). Finally, the supernatant was ultracentrifuged (Ultracentrifuge, Beckman Coulter, USA) at 100,000 g for 70 min to collect the OMVs pellet, which was additionally washed with phosphate-buffered saline (Gibco, MA, USA) at 100,000 g for 70 min to eliminate protein contamination. The pre-processed OMVs were either fresh or stored at −80°C until use.

### Nano flow cytometry

Nano-flow cytometry (NFC) was used to analyze the particle concentration and size distribution of OMVs samples using the NanoFCM instrument (NanoFCM, Xiamen, China) [23,24]. In brief, single-particle side scatter (SSC) and fluorescence were detected using two single-photon counting avalanche photodiodes (APDs). The particle concentration was calibrated using 200 nm PE and AF488 fluorescent bead conjugates, and the size distribution was calibrated using a cocktail of silica nanoparticles (NanoFCM Inc., S16M-Exo). All samples were diluted to achieve an optimal range of particle count between 2,000 to 12,000/min. Calibration curves were used to convert flow rate and SSC intensity into corresponding vesicle concentration and size using the NanoFCM software (NanoFCM professional V1.0).

### Transmission electron microscopy

Evaluation of isolated exosomes was performed using transmission electron microscopy (TEM). First, 10 μL of each sample was dropped onto a 200-mesh ultrathin carbon-coated copper grid for 2 min and then quickly dried on filter paper. The grid was negatively stained twice with 1% uranyl acetate (filtered through a 0.22 μm filter). After complete drying for 1 min, images were acquired using a Hitachi HT-7700 TEM at 100 kV.

### Coomassie Blue staining

SDS–PAGE was performed to resolve proteins from *P. gingivalis* and *P.g*-OMVs. Briefly, 10 μg of protein extract from each sample was loaded per lane and separated by electrophoresis. Gels were subsequently stained with Coomassie Brilliant Blue (Beyotime, Shanghai, China) for 20 min and imaged using a ChemiDoc MP system (Bio-Rad, USA).

### BMSCs culture

The mouse BMSCs were purchased from Cyagen Biosciences Technology (MUBMX-01001, Guangzhou, China) and cultured in Dulbecco’s modified Eagle’s medium (DMEM; Gibco, MA, USA) containing 10% fetal bovine serum (FBS; Gibco, MA, USA) and 10% penicillin/streptomycin (Gibco, MA, USA). For osteogenic differentiation studies, BMSCs were cultured in osteogenic induction medium (OM) consisting of complete medium supplemented with 50 μg/mL L-ascorbic acid (Sigma Aldrich, St. Louis, MO, USA), 10 mM β-glycerophosphate (Sigma Aldrich, St. Louis, MO, USA), and 10 nM dexamethasone (Sigma Aldrich, St. Louis, MO, USA).

### *P.g*-OMVs internalization in BMSCs

*P.g-*OMVs were labeled with PKH-26 (Sigma-Aldrich, St. Louis, MO, USA) to determine their uptake by BMSCs. *P.g-*OMVs were diluted in 1 mL diluent C, and 4 µL PKH26 dye diluted in 1 mL diluent C were incubated together. After 4 min, 2 mL 0.5% BSA/PBS was added to bind the excess dye for 5 min. Then, labeled OMVs were washed in PBS at 200,000 g for 1 h. After that, the labeled OMVs were co-cultured with BMSCs for 4 h. After the incubation, cells were washed twice with PBS and stained with Hoechst (Sigma-Aldrich, St. Louis, MO, USA) for 1 min. Images were captured using a Leica TCS-SP8 confocal imaging system (LEICA, Germany).

### CCK-8 viability assays

The BMSCs (3000 cells/well) were seeded into a 96-well plate and treated with *P.g*-OMVs (5 and 10 µg protein/mL) for 24 and 72 h. Cells were washed with PBS and incubated with a 110 µL fresh medium containing a 10 µL Cell Counting Kit (CCK)-8 solution (Dojindo Corp., Japan) for 3 h. Cell viability was determined by measurement of the absorbance using a spectrophotometer at a wavelength of 450 nm.

### Colony-forming unit assays

The BMSCs (200 cells/well) were seeded into a 6-well plate, and cell isolation was ensured to be completely separated. After overnight incubation, cells were treated with 5 or 10 μg/mL of *P.g*-OMVs or left untreated as a control. The medium was changed every 2 days, and after 7 days, the supernatant was removed, and the cells were washed gently with PBS 2–3 times. Then, the cells were fixed with 4% paraformaldehyde for 15 min, stained with crystal violet for 20 min, and observed and photographed after washing away the staining solution.

### EdU staining

The proliferation of BMSCs after 5 or 10 μg/mL of *P.g*-OMVs treatment was evaluated using the BeyoClick™ EdU Cell Proliferation Assay Kit (Beyotime, Shanghai, China), according to the manufacturer’s protocol (http://www.beyotime.com/product/C0085S.htm). After completion of the EdU staining, the cells were subjected to double staining with eosin staining solution (Beyotime, Shanghai, China) and DAPI staining solution (Beyotime, Shanghai, China), followed by photographing and quantitative analysis using Imagepro-Plus analysis software.

### Alkaline phosphatase (ALP) staining and ALP activity assay

For osteogenic differentiation studies, the osteogenic induction medium (BMSCs complete growth medium with 50 μg/mL L-ascorbic acid (Sigma Aldrich, St. Louis, MO, USA), 10 mM β-glycerophosphate (Sigma Aldrich, St. Louis, MO, USA), and 10 nmol/L dexamethasone (Sigma Aldrich, St. Louis, MO, USA) was added in the cultures. The BMSCs (2.5 × 10^4^ cells/well) were seeded in 48-well plates. ALP activity was determined on days 4 using an ALP kit according to the manufacturer’s protocol (Nanjing Jiancheng Bioengineering Institute, Nanjing, China) and normalized to total protein content. Total protein was measured by a commercial BCA protein assay kit (Beyotime Institute of Biotechnology, Shanghai, China). According to the manufacturer’s instructions, the ALP staining was performed using a BCIP/NBT alkaline phosphatase color development kit (Beyotime Institute of Biotechnology, Shanghai, China)

### Alizarin red S (ARS) staining

Cells cultured in 48-well plates were stained with 2% ARS solution (pH 4.2) on days 14 to visualize the mineralized matrix in the BMSCs culture. Images were obtained with a stereomicroscope (Leica, Singapore). To quantify the mineralized matrix, the ARS-stained calcium deposition was extracted with 10% cetylpyridinium chloride (CPC, Sigma Aldrich, St. Louis, MO, USA) for 20 min, and the absorbance of the extract was measured at 562 nm wavelength in a microplate reader.

### RT-qPCR analysis

*P.g*-OMVs-induced changes in gene expression of BMSCs were analyzed using RT-qPCR. Total RNA was extracted using a Steady Pure Universal RNA Extraction Kit (Accurate Biology, Changsha, China) and reverse transcribed into cDNA using a PrimeScript RT reagent kit with gDNA Eraser (Takara, Dalian, China) according to the manufacturer’s protocols. The expression of Alp, Ocn, Runx2, Saa1, Saa2, and Saa3 was analyzed by RT-qPCR. The RT-qPCR was performed using the TB Green Premix Ex Taq II kit (Takara, Dalian, China). The conditions for the PCR reaction were 1 cycle of 95°C for 30 s, followed by 40 cycles of 95°C for 5 s and 60°C for 30 s. Each reaction was performed in triplicate. The 2^−ΔΔCT^ method was used to calculate the relative expression of mRNA levels. The relative mRNA expression levels were standardized to the levels of the reference gene GAPDH. The primer sequences for the tested genes are listed in Table 1.

**Table 1.** Primers used for RT-qPCR analysis.

Gene	Acc No.	Primer Sequence (5’- > 3’)	Product Length (bp)
*Mus-Gapdh*	NM_001411843.1	F: TGTGTCCGTCGTGGATCTG R: TTGCTGTTGAAGTCGCAGGA	150
*Mus-Alp*	NM_007431.3	F: TGCCTACTTGTGTGGCGTGAA R: TCACCCGAGTGGTAGTCACAATG	164
*Mus-Ocn*	NM_007541.3	F: AGCAGCTTGGCCCAGACCTA R: TAGCGCCGGAGTCTGTTCACTAC	178
*Mus-Runx2*	NM_001271630.2	F: CACTGGCGGTGCAACAAGA R: TTTCATAACAGCGGAGGCATTTC	144
*Mus-Saa1*	NM_009117.4	F: TTTGTTCACGAGGCTTTCC R: CCTTTGAGCAGCATCATAGTT	126
*Mus-Saa2*	NM_011314.3	F: TGGCTGGAAAGATGGAGACAA R: AAAGCTCTCTCTTGCATCACTG	115
*Mus-Saa3*	NM_011315.3	F: AGAGAGGCTGTTCAGAAGTTCA R: AGCAGGTCGGAAGTGGTTG	111

### Western blot analysis

RIPA buffer (Beyotime, Beijing, China) supplemented with a protease inhibitor cocktail (Sigma, MO, USA) was used to lyse cells. Protein extracts were separated by SDS-PAGE and transferred to a PVDF membrane (Millipore, MA, USA). The membranes were blocked with QuickBlock blocking buffer (Beyotime, Beijing, China) and incubated with primary antibodies against GAPDH (ab181602, Abcam, USA), MyD88 (ab133739, Abcam, USA), TLR4 (ab13556, Abcam, USA), TLR2 (ab209217, Abcam, USA), SAA3 (ab233547, Abcam, USA), OCN (ab93876, Abcam, USA), NF-κB p65 (8242, CST, USA) overnight at 4°C. The specific secondary antibodies used were goat anti-rabbit and goat anti-mouse secondary antibodies. The protein bands were detected and quantified using a BioAnaly imaging system (BioAnaly, China).

### RNA sequencing

Total RNA from BMSCs undergoing osteogenic differentiation with or without *P.g*-OMVs treatment for 4 days was isolated using the RNeasy Mini Kit (Qiagen, Germany). Following the manufacturer’s instructions, paired-end libraries were prepared using the ABclonal mRNA-seq Lib Prep Kit (ABclonal, China). The library quality was assessed using the Agilent Bioanalyzer 4150. Subsequently, Illumina NovaSeq 6000 sequencing was performed to generate 150bp paired-end reads. The resulting clean reads were aligned separately to the reference genome using the HISAT2 software (http://daehwankimlab.github.io/hisat2/) in orientation mode to obtain mapped reads. Feature counts (http://subread.sourceforge.net/) counted the number of reads mapped to each gene. Subsequently, the FPKM (fragments per kilobase of exon per million fragments mapped) for each gene was calculated based on the gene length and the reads count mapped to that gene. Differential expression analysis was performed using the DESeq2 (http://bioconductor.org/packages/release/bioc/html/DESeq2.html). differentially expressed genes (DEGs) with |log2FC | > 1 and Padj < 0.05 were considered to be significantly different expressed genes. After obtaining differential expression of target mRNA reads, we performed a Gene Ontology (GO) enrichment analysis (http://www.geneontology.org) and a Kyoto Encyclopedia of Genes and Genomes (KEGG) pathway analysis (http://www.genome.jp/kegg) on the top 50 significantly DEGs following treatment with *P.g*-OMVs.

### SAA3 knockdown in BMSCs

Lentiviruses targeting Saa3 (sh-Saa3) and negative control (NC) vectors (sh-NC) were purchased from GenePharma Co., Ltd. (Suzhou, China). Specific interference sequences, shSaa3–63: 5’-GCATCTTGATCCTGGGAGTTG-3’, shSaa3–120: 5’-GTCAAGGGTCTAGAGACATGT-3’, shSaa3–320: 5’-GCTGACCAGTTTGCCAATGAG-3’ were used. Lentiviral infection was performed according to the manufacturer’s instructions (MOI = 50).

### Statistical analysis

All quantitative data are presented as mean values ± standard deviation (SD) for *n* ≥ 3 (number of experiments). Statistical analyses were performed using GraphPad Prism (version 10.2, GraphPad Software, La Jolla, CA, USA). A one-way analysis of variance (ANOVA) followed by Bonferroni’s multiple comparisons test was performed for comparisons involving more than two groups. An unpaired t-test was used for comparison of two groups. A p-value < 0.05 was considered statistically significant.

## Results

### P.g-OMVs isolation and characterization

**Figure 1.**
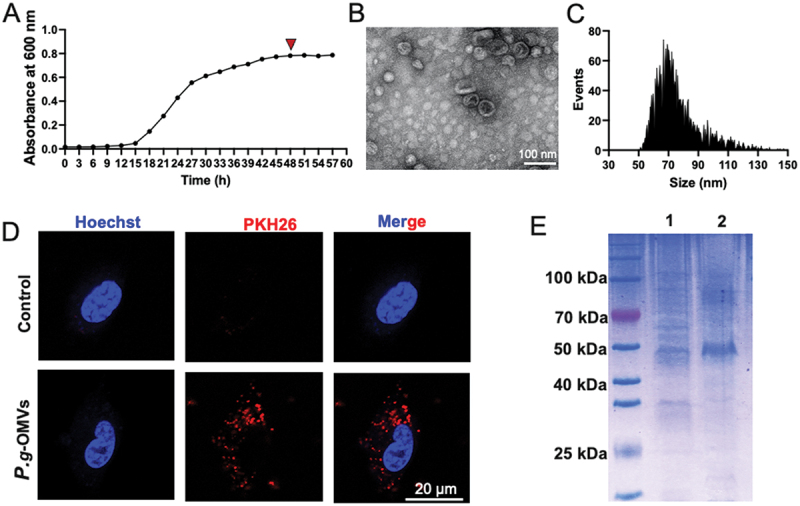
Purification and identification of *P.g*-OMVs. (A) The *P. gingivalis* growth until log phase (48 h). (B) TEM images of *P.g*-OMVs isolated from the conditioned medium at 48 h of culture *P. gingivalis*. (C) High sensitivity flow cytometry analysis of *P.g*-OMVs. (D) Fluorescence images of *P.g*-OMVs in BMSCs. (E) Coomassie blue protein staining of the *P. gingivalis* proteins (channel 1) and *P.g*-OMVs proteins (channel 2).

*P. gingivalis* reached the stationary phase at 48 h (Figure 1A), after which the conditioned medium was collected for the isolation of *P.g*-OMVs via ultracentrifugation. TEM revealed that *P.g*-OMVs were spherical bilayered fragments (Figure 1B), with an average diameter of 76.10 ± 15.16 nm, as determined by NFC (Figure 1C). Confocal microscopy confirmed that PKH26-labeled *P.g*-OMVs were internalized by BMSCs within a period of 4 h (Figure 1D). Coomassie blue staining indicated that *P.g*-OMVs exhibited higher protein enrichment in the heavy chain region (~50 kDa) compared to whole *P. gingivalis* cells (Figure 1E). These findings validate the successful isolation of *P.g*-OMVs and their subsequent uptake by BMSCs.

### *P.g*-OMVs promoted BMSCs’ proliferation

**Figure 2.**
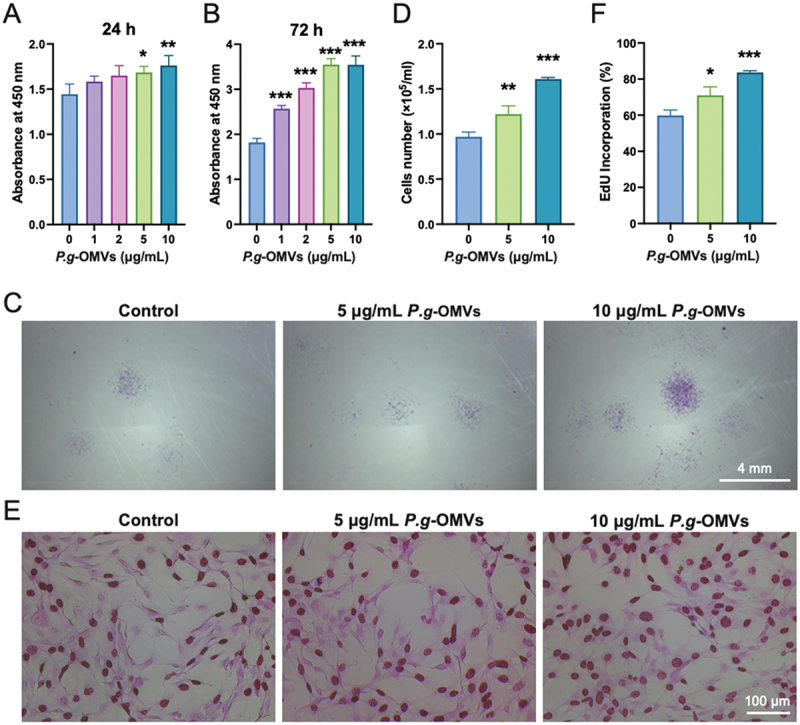
*P.g*-OMVs promoted BMSCs’ proliferation. (A, B) Cell viability was measured using CCK-8 assay at 24 and 72 h, *n* = 4. (C, D) Crystal violet staining for cell colony formation and colony quantification, *n* = 3. (E, F) EdU cell proliferation assay and quantification, *n* = 3. Significant difference compared with 10 µg/mL *P.g*-OMVs group, *p < 0.05, **p < 0.01, and ***p < 0.001.

*P.g*-OMVs were found to enhance the viability of BMSCs in a dose- and time-dependent manner within a concentration range of 1–10 μg/mL, as determined by CCK-8 assay results (Figure 2A,B). The colony formation assay, which is a reliable method for evaluating the proliferative capacity of individual cells, demonstrated that treatment with 10 μg/mL of *P.g*-OMVs significantly increased the proliferation of individual BMSCs (Figure 2C). Compared with the group without *P.g*-OMVs, the number of cells increased by 60% with 10 μg/mL *P.g*-OMVs (Figure 2D). EdU staining revealed increased newly synthesized DNA, as indicated by a higher percentage of EdU-positive cells following *P.g*-OMVs treatment (Figure 2E,F). According to these results, *P.g*-OMVs promote the proliferation of BMSCs.

### *P.g*-OMVs inhibit the osteogenic differentiation in BMSCs

**Figure 3.**
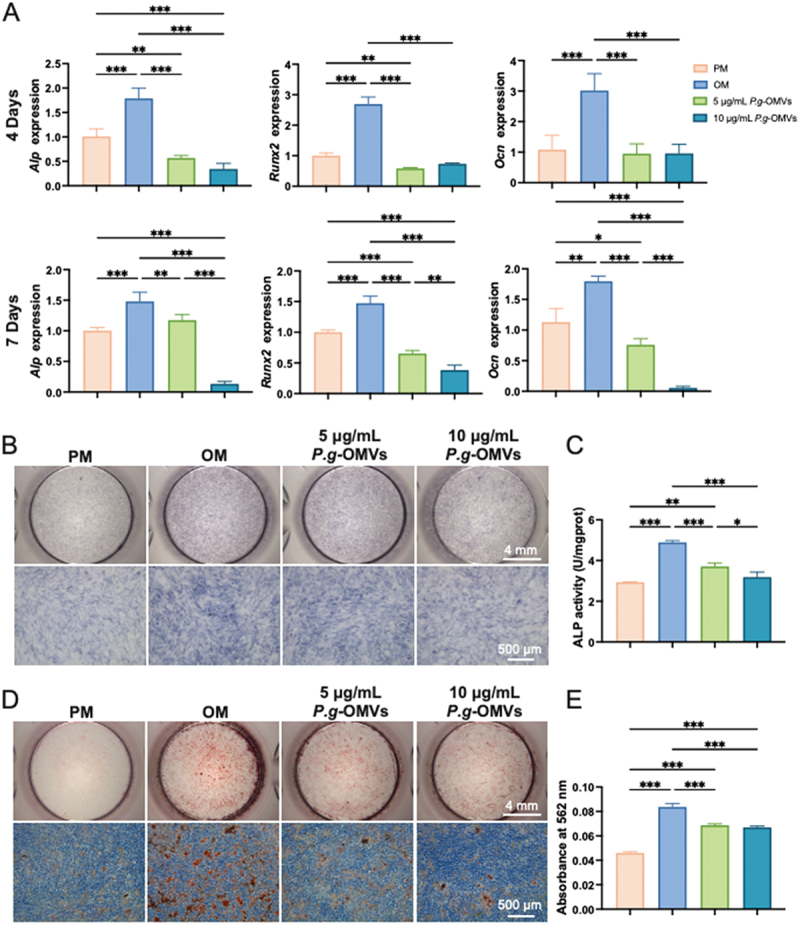
*P.g*-OMVs inhibited osteogenic differentiation of BMSCs. (A) The osteogenic genes (Alp, Ocn, and Runx2) in BMSCs induced by *P.g*-OMVs for 4 and 7 days, *n* = 4. (B) Cells were cultured in the absence or presence of *P.g*-OMVs. On day 4, cells were subjected to ALP staining. (C) The activity of ALP in BMSCs induced by *P.g-*OMVs for 4 days, *n* = 3. (D) The calcific nodules of the extracellular matrix were detected by alizarin red staining after 14 days of osteogenic induction. (E) Quantitative analysis of mineralized matrix in BMSCs culture, *n* = 3. Data were analyzed with one-way ANOVA with Bonferroni’s multiple comparison test. PM, proliferation medium; OM, osteogenic induction medium. *p < 0.05, **p < 0.01, and ***p < 0.001.

RT-qPCR analysis demonstrated a significant reduction in the expression of Alp, Runx2, and Ocn in BMSCs cultured with *P.g*-OMVs on days 4 and 7. Furthermore, a higher concentration of *P.g*-OMVs resulted in a more pronounced decrease in the expression of these genes (Figure 3A). *P.g*-OMVs inhibited ALP production and activity during 4 days of BMSCs culture (Figure 3B,C). ARS staining revealed that *P.g*-OMVs also inhibit matrix mineralization in BMSCs during 14 days culture. 14 (Figure 3D,E). These findings suggest that *P.g*-OMVs inhibit the osteogenic differentiation of BMSCs. Following these findings, the optimal dose of *P.g*-OMVs for subsequent experiments was 10 μg/mL.

SAA3 was markedly increased in BMSCs undergoing osteogenic differentiation and treated with P.g-OMVs

**Figure 4.**
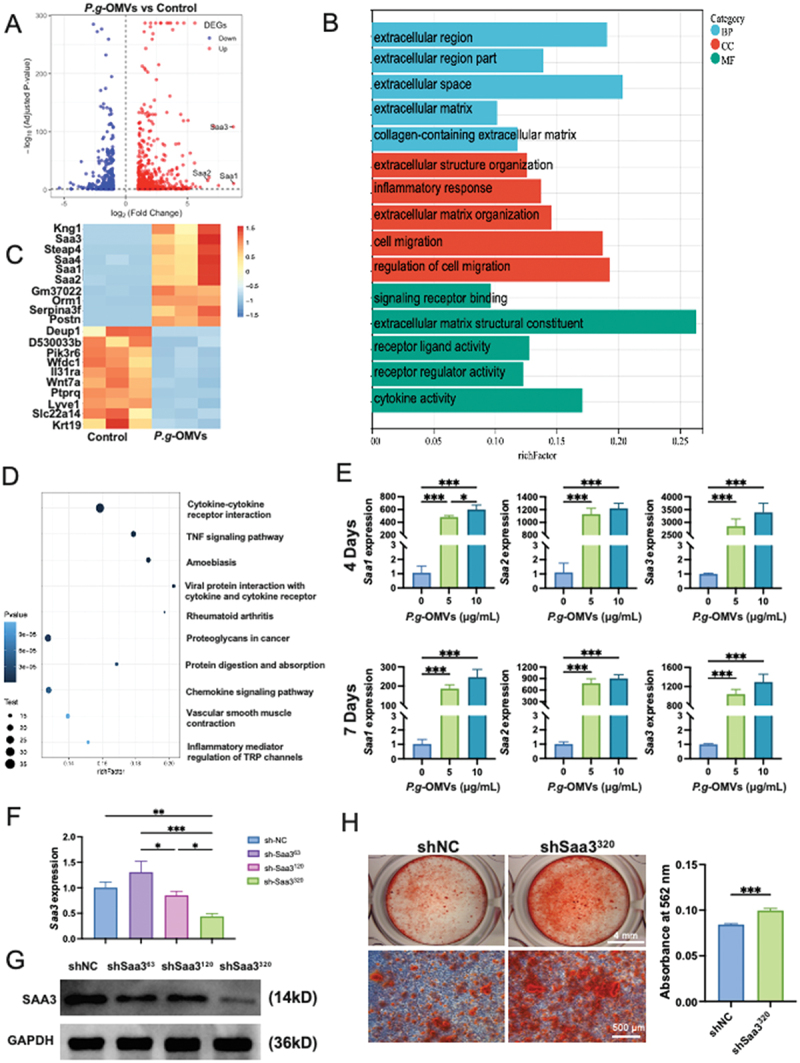
*P.g*-OMVs significantly induce the high expression of *Saa3* in BMSCs during osteogenic differentiation. (A) Volcano plot of differentially expressed genes (DEGs) in *P.g*-OMVs-treated BMSCs. (*n* = 3 per group). (B) Gene ontology (GO) enrichment analysis histograms and chord diagrams of DEGs in P.g-OMVs-treated BMSCs. (C) Heatmaps of the top 10 upregulated and downregulated genes in P.g-OMVs-treated versus untreated BMSCs. (D) KEGG analysis of DEGs was carried out, and 10 significant enrichment pathways are shown. (E) The SAA family genes (Saa1, Saa2, and Saa3) expression in BMSCs induced by *P.g*-OMVs for 4 and 7 days. (F, G) RT-qPCR and western blot were used to detect the inhibition effect of shRNA at different sites on SAA3 in BMSCs. (H) Knockdown of SAA3 expression in BMSCs enhanced the inhibition of osteogenesis caused by *P.g*-OMVs. *n* = 3, data were analyzed with one-way ANOVA with Bonferroni’s multiple comparison test. *p < 0.05, **p < 0.01, and ***p < 0.001.

RNA-seq analysis revealed that 335 genes were upregulated and 143 were downregulated in BMSCs treated with *P.g*-OMVs compared to the untreated control (Figure 4A). GO functional analysis showed that the ‘signaling receptor binding’ pathway was enriched in the Molecular Function (MF) category (Figure 4B). The SAA family genes (Saa1–4) were confirmed among the top 10 upregulated genes after *P.g*-OMVs treatment (Figure 4C). The top 10 KEGG pathways showed significant enrichment in the ‘TNF signaling pathway’ (Figure 4D). RT-qPCR indicated that following treatment with *P.g*-OMVs on the 4th day, Saa1 increased approximately 600-fold, Saa2 increased approximately 1200-fold, and Saa3 increased approximately 3100-fold (Saa4 was undetectable). On the seventh day, Saa1 increased approximately 230-fold, Saa2 increased approximately 800-fold, and Saa3 increased approximately 1200-fold (Figure 4E). Furthermore, knockdown of SAA3 restored matrix mineralization in *P.g*-OMVs-treated BMSCs (Figure 4H) by inhibiting the expression of SAA3 (Figure 4F,G) (Supplementary Figure S1). These results indicated that SAA family members, especially SAA3, may play a crucial role in *P.g*-OMVs-induced inhibition of osteogenic differentiation of BMSCs.

### TLR4 serves as the primary receptor for SAA3-mediated inhibition of osteogenic differentiation

**Figure 5.**
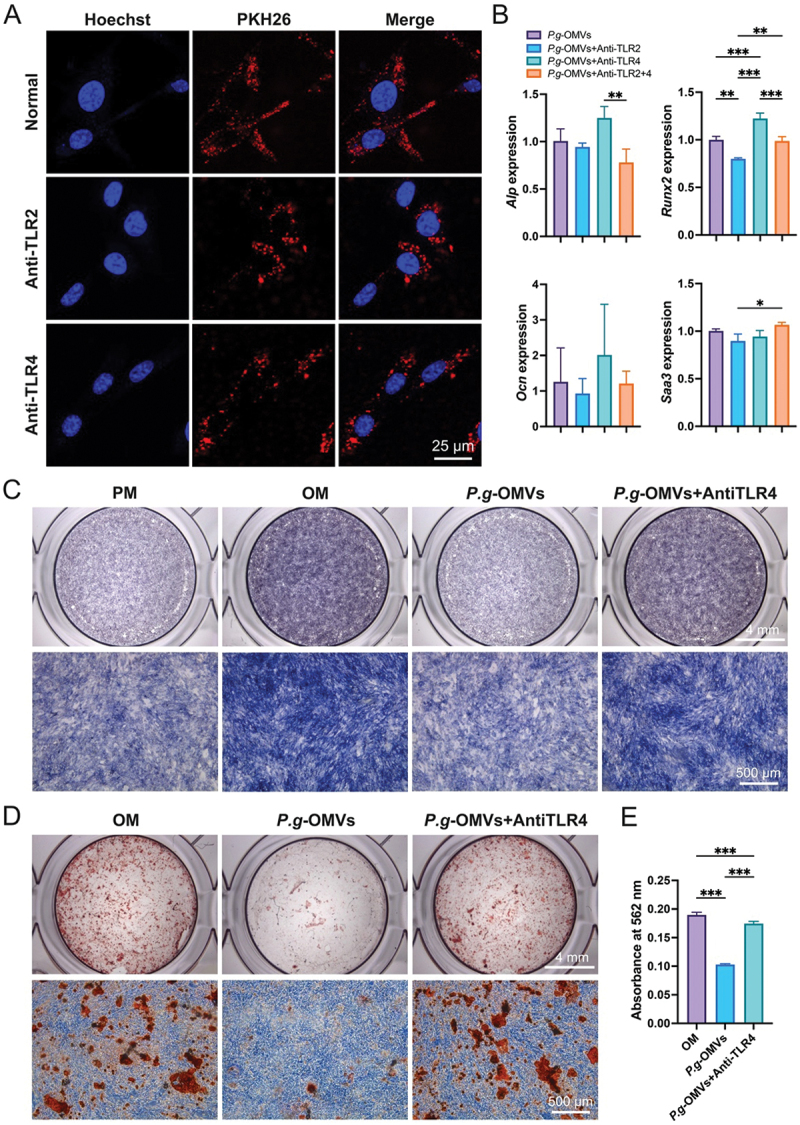
TLR4 serves as the primary receptor for SAA3-mediated inhibition of osteogenic differentiation. (A) Cellular internalization of *P.g*-OMVs into BMSCs under block TLR2 or TLR4, BMSCs were incubated with PKH-26 labeled *P.g*-OMVs (red) for 4 h. The nucleus of BMSC was stained with Hoechst (blue). (B) Alp, Runx2, Ocn, and Saa3 expression in BMSCs on the 4 days of osteogenic differentiation under block TLR2 or TLR4. (C) Cells were cultured in the absence or presence of *P.g*-OMVs with or without blocking TLR4. On day 4, cells were subjected to ALP staining. (D) The calcified nodules of the extracellular matrix were detected by alizarin red staining after 14 days of with or without block TLR4 osteogenic induction. (E) Quantitative analysis of mineralized matrix in BMSCs culture. *n* = 3, data were analyzed with one-way ANOVA with Bonferroni’s multiple comparison test. PM, proliferation medium; OM, osteogenic induction medium. ***p < 0.001.

As part of the innate immune response, toll-like receptors (TLRs) sense pathogen- or damage-associated molecular patterns [25]. SAA is known to be mediated through TLR2 and TLR4 receptors [18]. Here, we identified the central downstream receptors for SAA3 in regulating osteogenic differentiation of BMSCs. Firstly, we employed TLR2 and TLR4 antibodies (0.5 mg/mL) to block the function of TLR2 and TLR4 in BMSCs, aiming to investigate whether TLR2 and TLR4 impact the internalization of *P.g*-OMVs by cells. The results showed that the loss of functionality of either TLR2 or TLR4 did not affect the entry of *P.g*-OMVs into BMSCs (Figure 5A). Next, BMSCs were examined on the fourth day after osteogenic induction with *P.g*-OMVs after blocking TLR2 or TLR4. RT-qPCR results revealed that the blockade of TLR2 or TLR4 did not significantly affect the expression levels of Saa3 compared to the group treated solely with *P.g*-OMVs. However, the expression of Runx2 and Alp increased only upon TLR4 blockade (Figure 5B), indicating that TLR4 serves as a significant downstream receptor for SAA3-mediated inhibition of osteogenic differentiation in BMSCs. We predicted the interactions between SAA3 and TLR4 using computational modeling, and the data confirmed that TLR4 is regulated by SAA3 (Supplementary Figure S2). Furthermore, we further validated that inhibition of the receptor TLR4 for SAA3 could restore the osteogenic suppression induced by *P.g*-OMVs, as evidenced by ALP (Figure 5C,D) and ARS staining (Figure 5E,F).

### *P.g*-OMVs regulate the osteogenic differentiation of BMSCs through the SAA3/TLR4/MyD88/NF-κB signaling pathway

**Figure 6.**
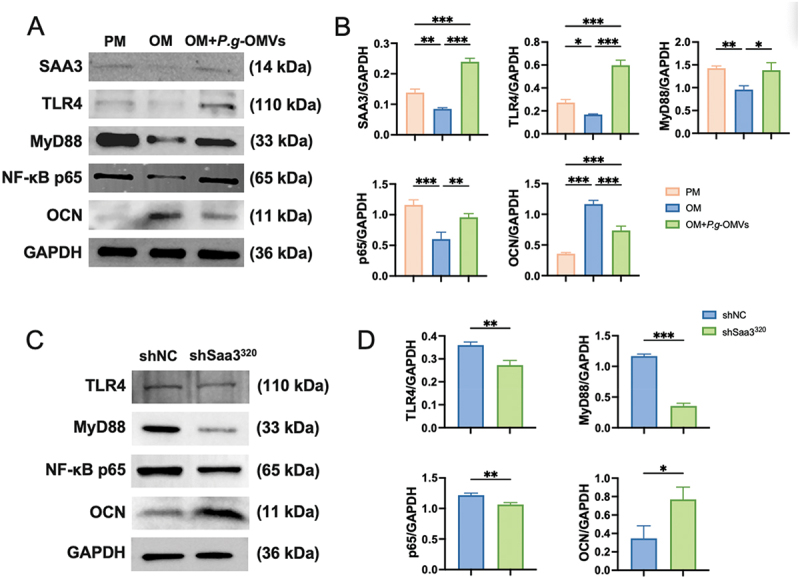
SAA3 inhibits osteogenic differentiation via the TLR4/MyD88/NF-κB signaling pathway of BMSCs. (A, B) Western blot was employed to investigate the influence of *P.g*-OMVs-induced SAA3 elevation on the TLR4/MyD88/NF-κB signaling pathway in BMSCs. (C, D) Knockdown of SAA3 expression in BMSCs enhanced the inhibition of osteogenesis caused by *P.g*-OMVs. *n* = 3, data were analyzed with one-way ANOVA with Bonferroni’s multiple comparison test. *p < 0.05, **p < 0.01, and ***p < 0.001.

TLR signalling pathways mediate NF-κB activation with the help of MyD88 [26]. Western blot results revealed that SAA3 protein expression was suppressed during the normal osteogenic differentiation of BMSCs on the fourth day. Conversely, *P.g*-OMVs (10 μg/mL) significantly upregulated SAA3 protein expression in osteogenically differentiating BMSCs. Similarly, the protein levels of TLR4, MyD88, and NF-κB p65 were downregulated during osteogenic differentiation but were significantly upregulated upon treatment with *P.g*-OMVs. *P.g*-OMVs also downregulated OCN protein expression in osteogenically differentiating BMSCs (Figure 6A,B) (Supplementary Figure S3). The results of western blot analysis showed that the knockdown of SAA3 downregulated the expression of TLR4, MyD88, and NF-κB p65 in *P.g*-OMVs-treated BMSCs (Figure 6C,D) (Supplementary Figure S4). These results collectively suggest that *P.g*-OMVs upregulate SAA3 expression in BMSCs and inhibit the osteogenic differentiation of BMSCs by regulating the TLR4/MyD88/NF-κB signaling pathway.

## Discussion

Bacterial OMVs carry bacterial byproducts and virulence factors and can quickly enter surrounding host cells. Bacterial OMVs also exert a systemic effect by entering circulation [27]. *P. gingivalis* is one of the critical pathogens related to periodontal diseases and alveolar bone loss [28]. *P.g*-OMVs have been reported to affect periodontal cells’ survival and functions and exert systemic effects [29]. However, the impact of *P.g*-OMVs on alveolar bone formation has not been investigated yet. Here, we unraveled that *P.g*-OMVs inhibit osteogenic differentiation of BMSCs. This effect was more pronounced than the effect of *P.g*-LPS. *P.g*-OMVs inhibited osteogenic differentiation of BMSCS mainly via SAA3-mediated upregulation of TLR4/MyD88/NF-κB p65 axis. Our results indicate that *P. gingivalis* exerts a catabolic effect on periodontal/alveolar bone via *P.g*-OMVs-mediated inhibition of the osteogenic differentiation of precursor cells.

*P. gingivalis* induces host immune responses and affects host cell functions through its secretion of LPS, fimbriae, gingipains, and other virulence factors [30]. *P.g*-OMVs contain the cargo of outer membrane and periplasmic proteins such as LPS, fimbriae, gingipains, and other virulence factors [31]. Previous studies have reported the catabolic effect of *P.g*-OMVs on macrophages, neuronal cells, endothelial cells, and intestinal cells [32–34]. Our results showed that *P.g*-OMVs promoted the proliferation of BMSCs. Sequencing results indicated that downregulation of Hippo signaling-related genes Wwc1 [35,36] and Llgl2 [37] in *P.g*-OMVs-treated BMSCs may induce proliferation.

**Figure 7.**
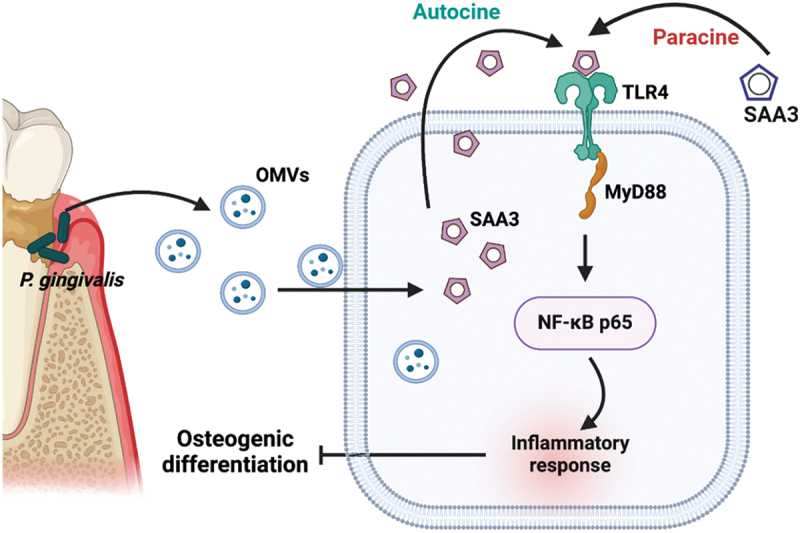
Scheme illustrating the inhibitory effect of *P.g*-OMVs in osteogenic differentiation of BMSCs via TLR4/MyD88/NF-κB signaling. in the context of periodontitis induced by *P. gingivalis*, there is an increase in the levels of free *P.g*-OMVs. These *P.g*-OMVs function as potent initiators, enhancing the expression and secretion of SAA3 in BMSCs. Upon binding to TLR4, SAA3 activates the NF-κB signaling pathway via MyD88, consequently inhibiting the osteogenic activity of BMSCs. The image was created with BioRender.com.

*P.g*-OMVs inhibited osteogenic differentiation of BMSCs. *P.g*-OMVs treatment upregulated SAA3 expression in BMSCs. Studies have shown elevated SAA levels in the saliva and serum of periodontal patients, with significantly higher levels in the gingival crevicular fluid of obese patients with periodontitis compared to those with only obesity or periodontitis [38,39]. SAA3 interacts with TLR2 and/or TLR4 to regulate post-infection immune responses [16,40]. Here, we found that *P.g*-OMVs-upregulated SAA3 interacts with TLR4. MyD88/NF-kB signaling is a key downstream target of TLR4 activation during infections [41,42]. Bacterial infection and LPS treatments have been reported to inhibit osteogenic differentiation of BMSCs via MyD88/NF-κB signaling [43,44]. Inhibition of SAA3 or TLR4 rescued *P.g*-OMVs-inhibited osteogenic differentiation of BMSCs. These results confirm that *P.g*-OMVs inhibit osteogenic differentiation of BMSCs via SAA3-mediated TLR4/MyD88/NF-kB signaling (Figure 7).

TLR4 can rapidly recognize microbial components, such as the LPS of Gram-negative bacteria, and initiate immune responses. Wild *P. gingivalis* evades TLR4 recognition and subsequent inflammasome activation via nonphosphorylated tetra-acylated lipid A (NPLA), thereby providing protection for the *in vivo* proliferation of *P. gingivalis* [45]. Recent studies have also demonstrated that *P.g*-OMVs can enhance the expression of TLR4, which is consistent with our findings. Additionally, *P.g*-OMVs are rich in C4′ monophosphoryl lipid A (C4′-MPLA), can function as an endogenous signal, directly interact with TLR4 and inflammasomes, and promote the downstream release of high levels of interferon-β (IFN-β) and interleukin-1β (IL-1β) [46]. IL-1β is regarded as the most potent inducer of SAA [19]. We analyzed the differences between *P.g-*LPS and *P.g*-OMVs during the osteogenesis of BMSCs. The results indicated that *P.g*-OMVs had a stronger inhibitory effect on osteogenic genes and a greater ability to upregulate SAA3 expression compared to *P.g-*LPS (Supplementary Figure S5). Apart from the NF-κB pathway, TLR4 can also be involved in multiple downstream signal transduction cascades, including the MAPK and IRF pathways. Studies have shown that *P.g*-OMVs can stimulate gingival epithelial cells to induce proinflammatory cytokines through the MAPK and stimulator of interferon genes (STING) pathways [11]. Therefore, whether *P.g*-OMVs have other osteogenesis-inhibitory pathways besides the TLR4/MyD88/NF-κB pathway remains to be investigated.

Although this study confirmed the inhibitory role of *P.g*-OMVs in osteogenic differentiation of BMSCs, some limitations do exist. We did not investigate the inhibitory role of *P.g*-OMVs in bone formation/regeneration using in situ models. Moreover, we did not examine the key factors of *P.g*-OMVs cargos responsible for inhibiting the osteogenic differentiation of BMSCs. Bone homeostasis is a complex process involving the activities of osteoblasts, osteoclasts, and osteocytes [47]. Therefore, the effects of *P.g*-OMVs on other bone cells, such as osteoclasts and osteocytes, need to be further investigated. Importantly, although our molecular model suggests the potential for an interaction between SAA3 and TLR4, additional validation through co-immunoprecipitation or other methods is necessary to confirm their direct interaction. The transient or context-dependent nature of this interaction might present technical challenges, which we aim to explore and address in our future studies.

## Conclusion

This study shows that *P.g-*OMVs promote proliferation, while they inhibited osteogenic differentiation of BMSCs. *P.g-*OMVs treatment robustly upregulated SAA3 during osteogenic differentiation of BMSCs. Here, we show that SAA3 binds with TLR4, activating the downstream MyD88/NF-κB signaling pathway, which inhibits the osteogenic differentiation of BMSCs. These results indicate that *P.g-*OMVs exert a catabolic effect on bone formation/regeneration that could contribute to periodontitis-induced alveolar bone loss. Our study suggests that targeting *P.g-*OMVs release or SAA3/TLR4/MyD88/NF-κB axis may be an effective strategy to rescue periodontitis-induced alveolar bone loss.

## Supplementary Material

Supplementary Figure.docx
