# Supplementary material for: Porphyromonas gingivalis OMVs inhibit osteogenic differentiation of BMSCs via SAA3/TLR4/MyD88/NF-κB axis
Source: J Oral Microbiol. 2025 Aug 8;17(1):2540823. doi: 10.1080/20002297.2025.2540823 (PMC12337729; doi:10.1080/20002297.2025.2540823)
Supplement: Supplementary Figure.docx [file ZJOM_A_2540823_SM1354.docx]

**Supplementary Figure S1**


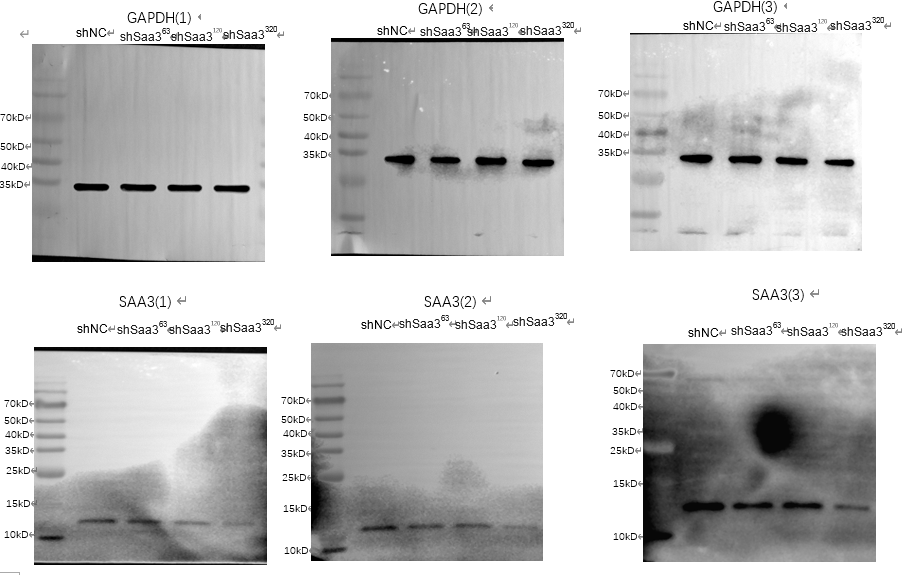


**Figure S1:** Original data of western blot analysis SAA3 and GAPDH in Figure 4G.

**Supplementary Figure S2**


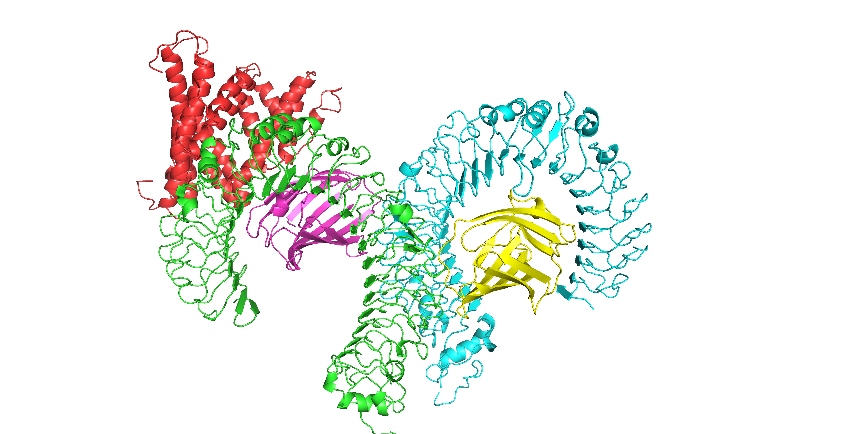

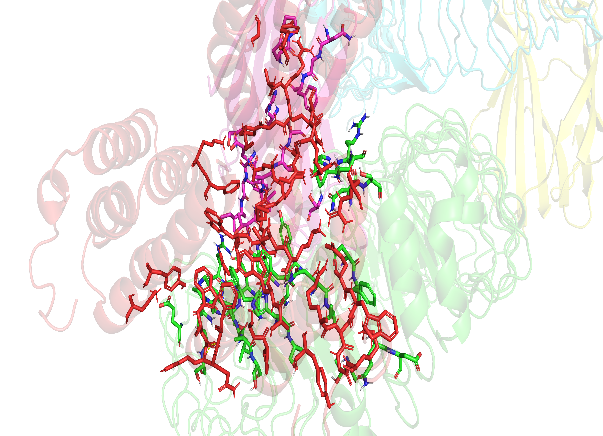


B

A

**Figure S2: Computer models predict SAA3 binding to TLR4.** (A) The red represents the SAA3 protein and the remaining colors represent the TLR4 protein. (B) At the binding site, red depicts the hydrogen bond of the amino acid of the SAA3 protein, while green represents that of the TLR4 protein.

**Supplementary Figure S3**


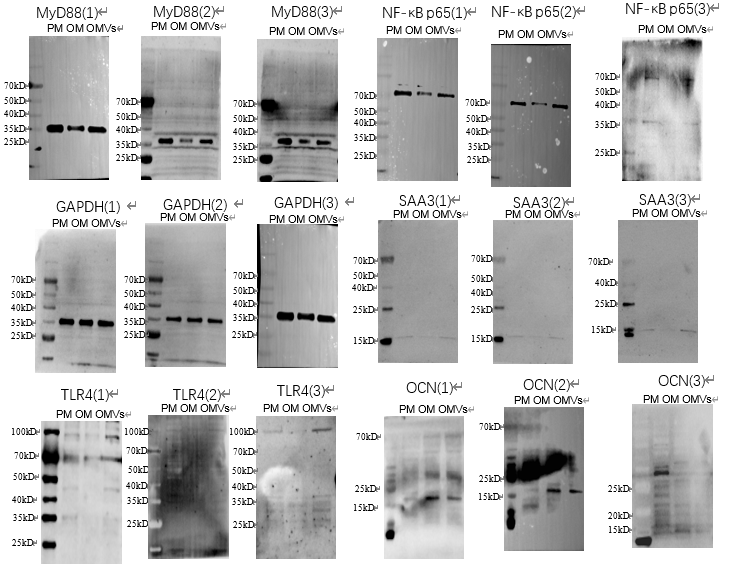


**Figure S2:**Original data of western blot analysis SAA3, TLR4, MyD88, NF-κB P65 ,OCN and GAPDH in Figure 6A and 6B.

**Supplementary Figure S4**


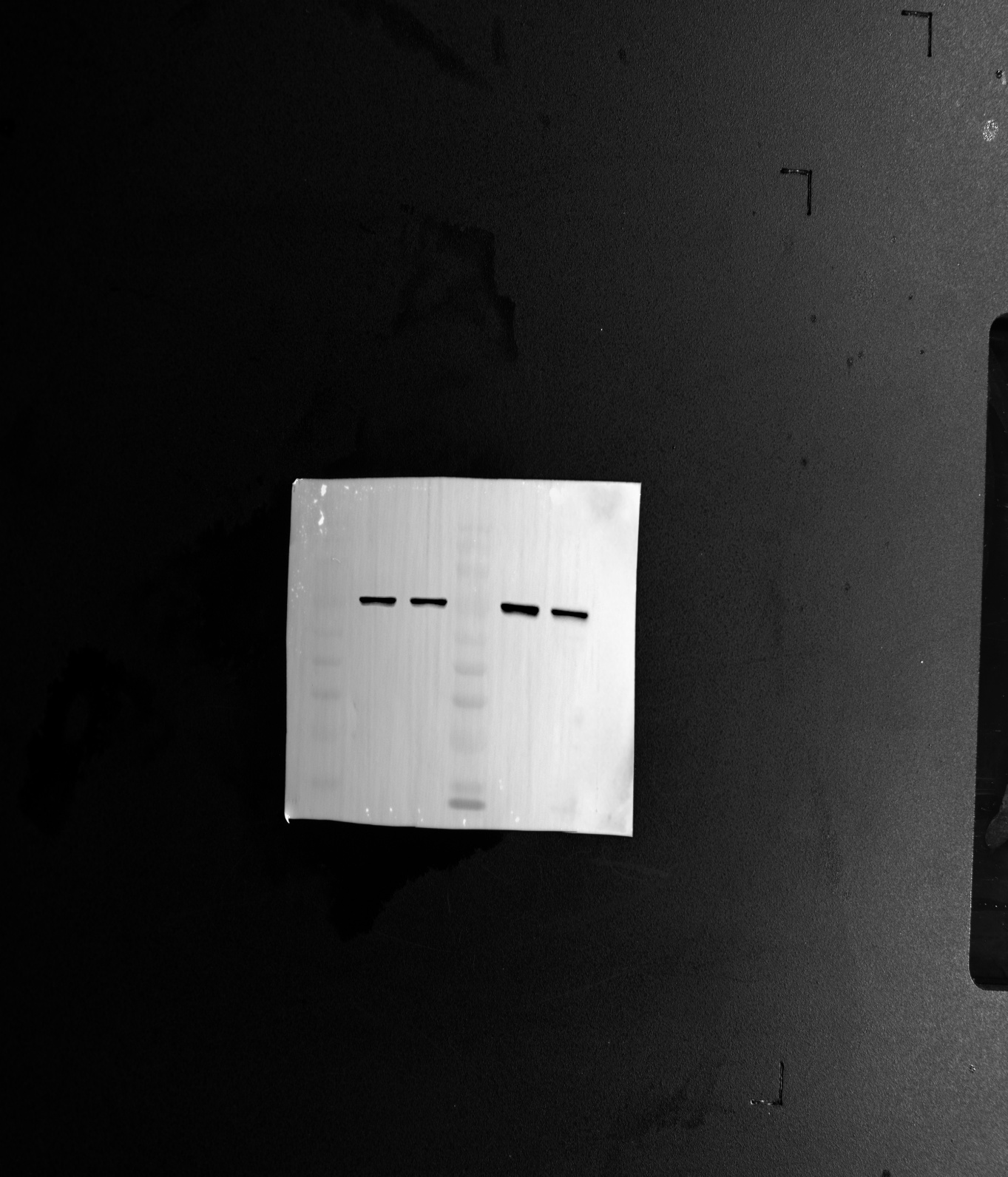


25kD

35kD

40kD

50kD

70kD

100kD

shSaa3^320^

P65（3）

shNC


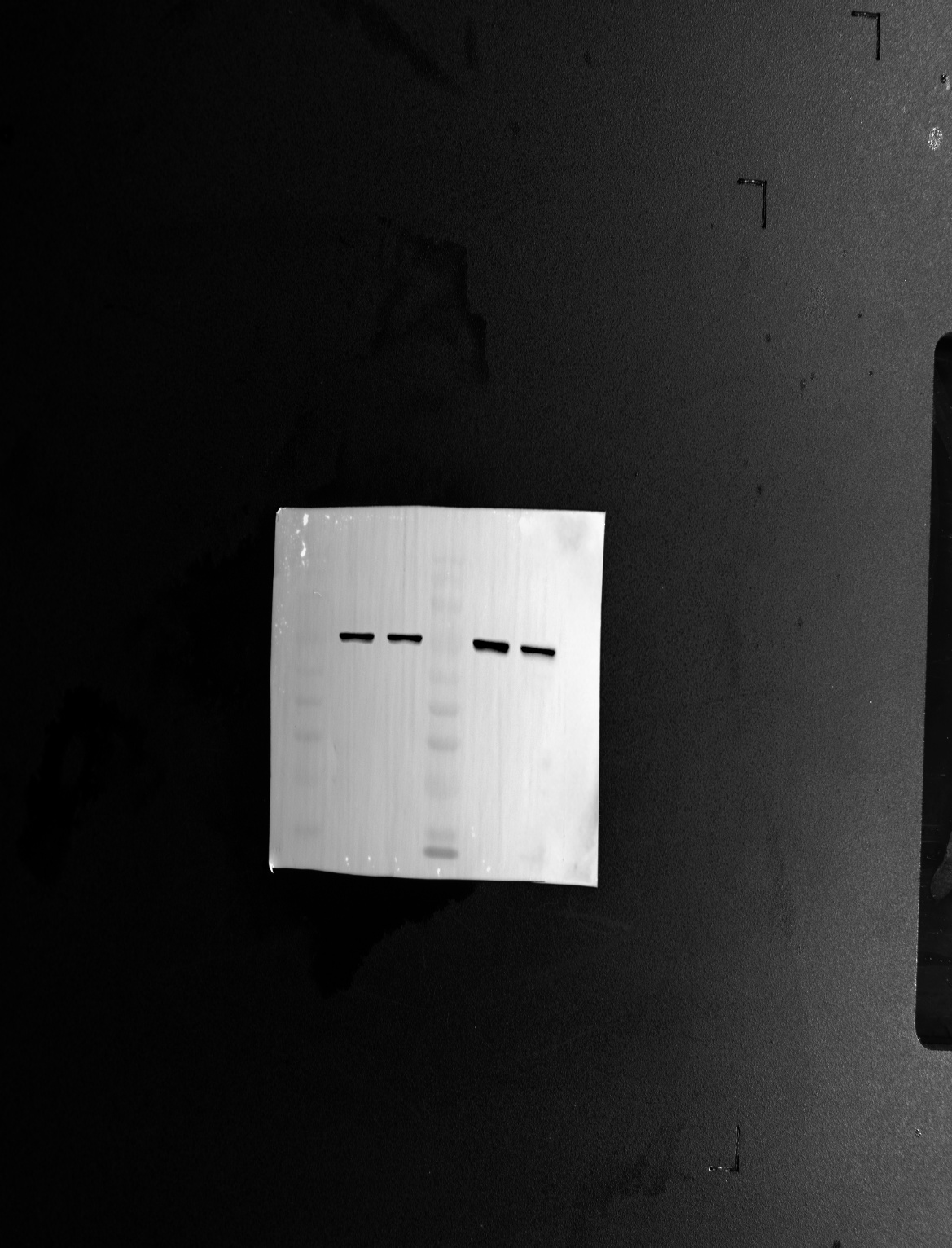


25kD

35kD

40kD

50kD

70kD

100kD

shSaa3^320^

P65（1）

shNC

25kD

35kD

40kD

50kD

70kD

100kD

shSaa3^320^

P65（2）

shNC


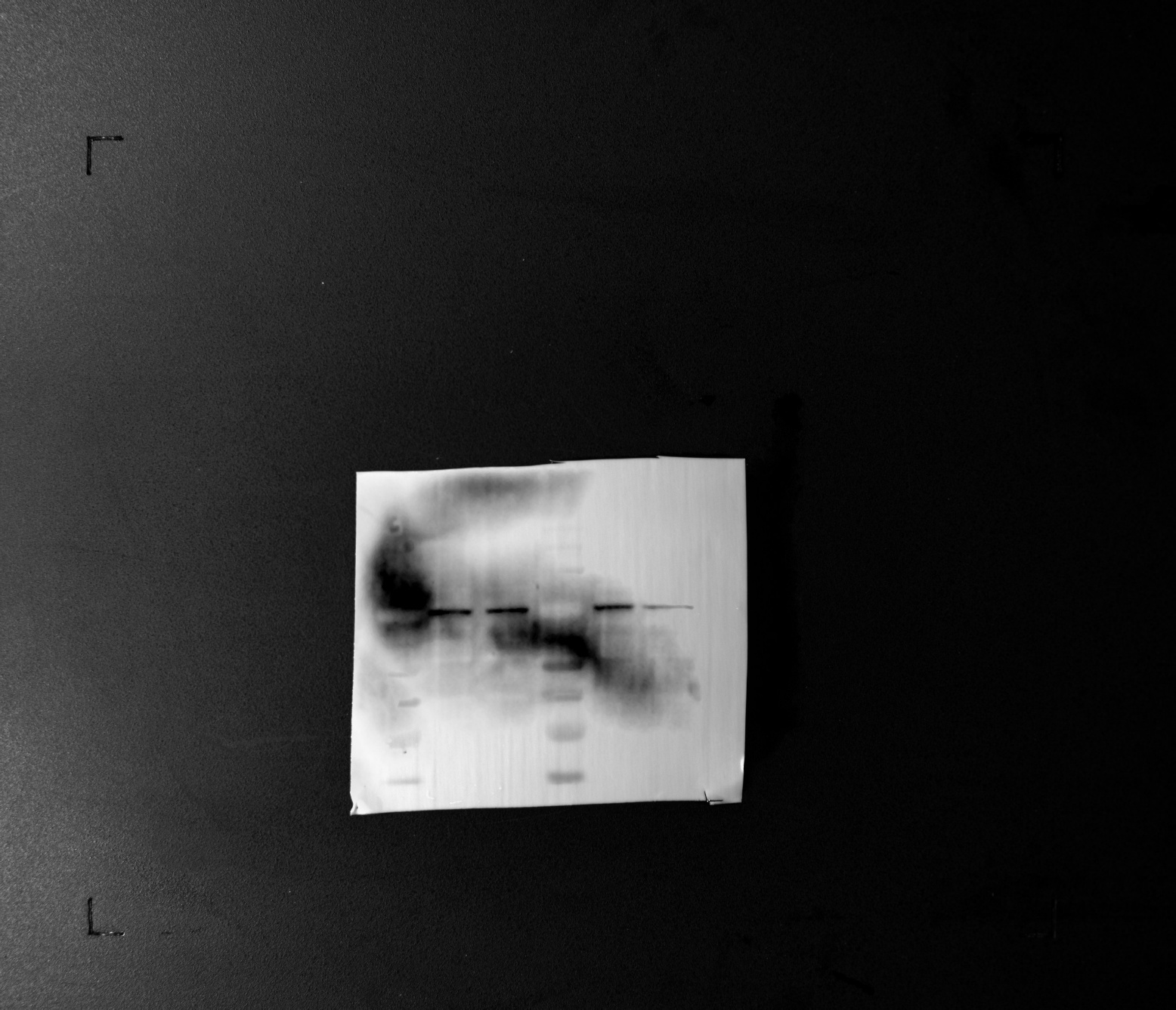


25kD

35kD

40kD

50kD

70kD

100kD

shSaa3^320^

GAPDH（1）

shNC


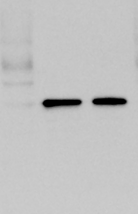


25kD

35kD

40kD

50kD

70kD

100kD

shSaa3^320^

GAPDH（2）

shNC


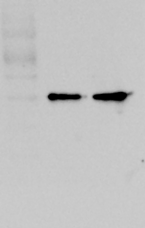


25kD

35kD

40kD

50kD

70kD

100kD

shSaa3^320^

GAPDH（3）

shNC


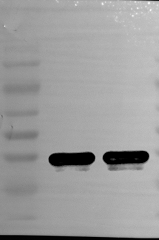


**Figure S4:** Original data of western blot analysis TLR4, MyD88, NF-κB P65, OCN and GAPDH in Figure 6C and 6D.


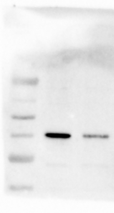


25kD

35kD

40kD

50kD

70kD

100kD

shSaa3^320^

MyD88（1）

shNC


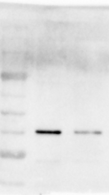


shSaa3^320^

MyD88（2）

shNC

shSaa3^320^

MyD88（3）

shNC


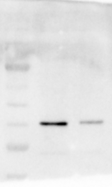


70kD

50kD

40kD

25kD

15kD

OCN(1)


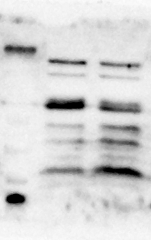


shSaa3^320^

shNC


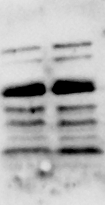


OCN(2)

shSaa3^320^

shNC


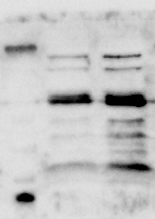


OCN(3)

shSaa3^320^

shNC


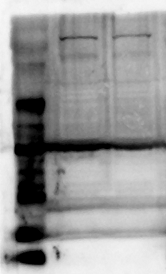


70kD

100kD

shSaa3^320^

TLR4（1）

shNC

TLR4（2）

shNC


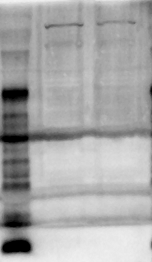

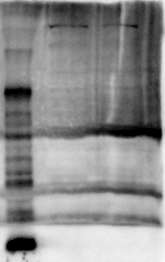


shSaa3^320^

TLR4（3）

shNC

shSaa3^320^


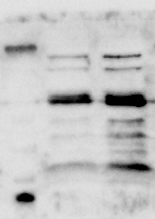


OCN(3)

shSaa3^320^

shNC

**Supplementary Figure S5**

**Figure S5:** Alp, Runx2, Ocn, and Saa3 expression in BMSCs on the 4 days and 7 days of osteogenic differentiation under treatment with 10 μg/mL *P.g*-LPS or *P.g*-OMVs，n = 3. Data were analyzed using one-way ANOVA with Bonferroni’s multiple comparison test. **p < 0.01 and ***p < 0.001 relative to *P.g*-LPS group
